# COMPREHENSIVENESS AND READABILITY OF CHATGPT’S ANSWERS TO DEMENTIA FAMILY CAREGIVERS’ QUESTIONS

**DOI:** 10.1093/geroni/igae098.3943

**Published:** 2024-12-31

**Authors:** Soojeong Han, Bayu Aryoyudanta, Yong Kyung Choi

**Affiliations:** Columbia University School of Nursing, New York, New York, United States; University of Pittsburgh, Pittsburgh, Pennsylvania, United States; University of Pittsburgh, Pittsburgh, Pennsylvania, United States

## Abstract

Family caregivers experience challenges in deciding on care transitions. As ChatGPT is a powerful tool for obtaining information, this study aims to evaluate the comprehensiveness and readability of ChatGPT’s answers to dementia family caregivers’ questions regarding transitioning to hospice and palliative care. We extracted data from a public online forum, ALZConnected.org, dedicated to caregivers and individuals affected by dementia. Our dataset comprised 35,482 original posts collected from 2011 to 2023. By filtering with the search terms “palliative”, “hospice”, or “end of life”, 123 questions regarding transitioning were discovered. Of 123 questions, 24 were selected with convenience sampling and entered into ChatGPT. The comprehensiveness of ChatGPT’s answers was evaluated using single-turn and then multi-turn conversations. The readability of all final answers was assessed using the Flesch Reading Ease (FRE) (Rouhi et al., 2024). Results revealed that 41.67 % (n = 10) was scored as comprehensive, 33.33 % (n = 8) as adequate, and 25.00 % (n = 6) as incomplete. The median comprehensiveness score was 2.00 (mean 2.08, SD 0.88). The mean (SD) FRE score was 25.37 (10.39), indicating ‘very difficult to read’. We further investigated whether ChatGPT can provide answers that are easier to read on a different literacy level. The mean (SD) FRE score of revised answers was 46.42 (12.99), indicating ‘difficult to read’. The t-test revealed significant differences between the two answers on the FRE scale (t = -6.20; p < 0.0001). Further studies can explore how to maximize the benefits of using ChatGPT for family caregivers.

